# Impact of Starting Knee Flexion Angle on Muscle Activity and Performance during Plyometrics without Jumping

**DOI:** 10.3390/s24010044

**Published:** 2023-12-20

**Authors:** Maximiliano Torres-Banduc, Ignacio Chirosa-Ríos, Luis Chirosa-Ríos, Daniel Jerez-Mayorga

**Affiliations:** 1 Escuela de Kinesiología, Facultad de Ciencias de la Salud, Universidad de Las Américas, Viña Del Mar 2520000, Chile; maxto22@gmail.com; 2 Department of Physical Education and Sports, Faculty of Sports Sciences, University of Granada, 18071 Granada, Spain; lchirosa@ugr.es; 3 Exercise and Rehabilitation Sciences Institute, School of Physical Therapy, Faculty of Rehabilitation Sciences, Universidad Andres Bello, Santiago 7570172, Chile; djerezmayorga@ugr.es

**Keywords:** plyometric, electromyography, dynamometry, limb dominance, physical training muscle strength

## Abstract

Most of the existing research has focused on jump plyometrics, where landing reaction forces must be dissipated among lower limb articulations. In contrast, the investigation of resisted plyometrics without jumping, devoid of such landing forces, remains relatively limited. This study aimed to (i) investigate the impact of resisted plyometrics without jumping at two knee flexion angles (60 and 90 degrees) on vastus muscle activity relative to limb dominance and (ii) assess strength, power, and work during the concentric–eccentric phases of these exercises. Thirty-one healthy participants underwent quantification of lower limb muscle amplitude, strength, power, and work during resisted plyometrics without jumping from both 60° and 90° knee flexion positions. After anthropometric evaluations, participants used a dynamometer with a load equal to 80% of body weight while wireless surface electromyography electrodes recorded data. Statistical analyses utilized paired t-tests or nonparametric equivalents and set significance at *p* ≤ 0.05. Results showed significantly higher muscle activity in the vastus medialis (VM) (dominant: 47.4%, *p* = 0.0008, *rs* = 0.90; nondominant: 54.8%, *p* = 0.047, *rs* = 0.88) and vastus lateralis (VL) (dominant: 46.9%, *p* = 0.0004, *rs* = 0.86; nondominant: 48.1%, *p* = 0.021, *rs* = 0.67) muscles when exercises started at 90° knee flexion, regardless of limb dominance. Substantial intermuscle differences occurred at both 60° (50.4%, *p* = 0.003, *rs* = 0.56) and 90° (54.8%, *p* = 0.005, *rs* = 0.62) knee flexion, favoring VM in the nondominant leg. Concentric and eccentric strength, power, and work metrics significantly increased when initiating exercises from a 90° position. In conclusion, commencing resisted plyometrics without jumping at a 90° knee flexion position increases VM and VL muscle activity, regardless of limb dominance. Furthermore, it enhances strength, power, and work, emphasizing the importance of knee flexion position customization for optimizing muscle engagement and functional performance.

## 1. Introduction

Plyometric training, characterized by the rapid stretching and contracting of muscles referred to as the stretch–shortening cycle (SSC), has been extensively studied for its efficacy in enhancing athletic performance and improving lower limb power. Performing plyometric exercises has the potential to enhance neuromuscular coordination, thereby improving neural efficiency. As a result, plyometric training can elevate neuromuscular performance and motor control, making neuromuscular coordination more automated [1]. Plyometric exercises enable muscles to generate force by enhancing the musculoskeletal system’s capacity to handle greater workloads without activating the Golgi tendon organ. Through plyometric training, neuromuscular coordination is enhanced by conditioning the nervous system, leading to the automation of movements during activities, known as the “training effect” [1]. This process reinforces motor patterns and automates actions, ultimately enhancing neural efficiency and boosting neuromuscular performance. However, most of the existing research has focused on jump plyometrics [2,3,4,5], where landing reaction forces must be dissipated among lower limb articulations. In contrast, the investigation of resisted plyometrics without jumping, devoid of such landing forces, remains relatively limited. In this context, exploring the effects of SSC to increase muscle performance during resisted plyometrics without jumping at different knee flexion angles becomes particularly relevant to identify optimal training strategies for athletes and active individuals. Starting at different knee angles can be beneficial for optimizing control, especially when the exercise is designed to target specific muscle groups [6]. Higher EMG activity at varying knee flexion angles may indicate increased activation or engagement of specific muscles at those angles. The usefulness of this information depends on the context. For instance, if the exercise aims to target these specific muscles, greater EMG activity can be advantageous. However, the choice of knee flexion angle should align with the training objectives and requirements. Therefore, it is essential to investigate the effects of different knee angles and movement phases in resisted nonjump plyometric exercises, not only from the perspective of EMG amplitude but also strength, power, and workload analysis.

A critical aspect often overlooked in plyometric studies is the consideration of lower limb dominance and the difference in muscle activity or performance between repeated concentric and eccentric phases. The dominant (D) limb, being more adept at generating force and power [7,8], may respond differently to training stimuli than the nondominant (ND) limb. This discrepancy in adaptation could lead to imbalances, potentially affecting performance and predisposing athletes to injury [9]. Examining the effects of plyometrics without jumping on lower limb muscular activity, and also in strength, power, and work during the concentric–eccentric phases with respect to limb dominance may provide valuable insights into how athletes respond individually to such training, ultimately guiding personalized training programs for improved performance outcomes.

Therefore, this study aimed to (i) investigate the effects of a resisted plyometric exercise without jumping at two knee flexion positions (60 and 90 degrees) on the muscle activity of the vastus muscles with regard to limb dominance and (ii) assess strength, power, and work during the concentric–eccentric phases of a resisted plyometric exercise without jumping. We hypothesize that in a resisted plyometric exercise without jumping, there will be significant differences in muscle activity of the vastus muscles between dominant and nondominant limbs at both 60 and 90 degrees of knee flexion. Additionally, we expect to find variations in strength, power, and work output during the concentric–eccentric phases between the two knee flexion positions, with one position potentially favoring greater performance metrics. These findings could have significant implications for athletes, coaches, and practitioners seeking to maximize the benefits of plyometric exercises while minimizing the risk of asymmetry and landing forces.

## 2. Materials and Methods

### 2.1. Experimental Design

In a cross-sectional study, an evaluation was conducted that encompassed the quantification of muscle amplitude, strength, power, and work levels in the lower limbs of a group of healthy subjects. This assessment was conducted during a resisted plyometric exercise without jumping, which involved two different starting positions: one at 60° and another at 90° of knee flexion. This experimental setup was designed to investigate the impact of the two knee flexion angles on the measured variables within each individual, employing a paired observation approach to enhance the precision of the analysis and minimize intersubject variability. After completing an anthropometric evaluation, participants underwent evaluation using a functional electromechanical dynamometer in tonic mode, with a load equivalent to 80% of their body weight. During these assessments, wireless surface electromyography electrodes were applied.

To enhance the reliability of the testing, a familiarization session was conducted. During this session, participants were provided with a detailed explanation of the exercise, and they performed three practice trials. Additionally, participants were instructed to refrain from engaging in physical training for a period of 24 h preceding the testing session.

### 2.2. Participants

The determination of the requisite sample size was performed through the utilization of statistical software (G*Power, v3.1.9.7, Heinrich-Heine-Universität, Germany). A medium effect size of 0.7, as ascertained from a previous investigation [9], was employed as a basis for this calculation. In light of the foregoing parameters, specifically a desired statistical power (1-ß error) of 0.95 and an alpha error threshold of less than 0.05, the total sample size was computed to be 25 participants. To account for potential attrition during the course of the study, the minimum sample size was established at 30 participants. The following inclusion criteria were enforced: (i) absence of musculoskeletal injuries within the preceding two months prior to the study commencement, (ii) no record of lower-extremity surgical procedures within the past year, and (iii) absence of any musculoskeletal conditions impeding the ability to engage in maximal effort during the testing protocols. A total of 31 healthy individuals (39% female; age: 23.1 ± 5.6 years; body mass: 71.93 ± 12.49 kg; height: 1.74 ± 0.09 m) participated in the investigation.

### 2.3. Data Recordings

#### 2.3.1. Anthropometric Assessment

Body mass was quantified using a calibrated mechanical scale (SECA model 711, Hamburg, Germany) with a precision level of 0.1 kg. Standing height was determined using a telescopic scale (SECA, model 220, Hamburg, Germany) with an accuracy of 0.1 cm. Participants were assessed while wearing lightweight attire, and footwear was excluded during the measurements.

#### 2.3.2. Resisted Plyometric without Jumping Evaluation

**Figure 1:**
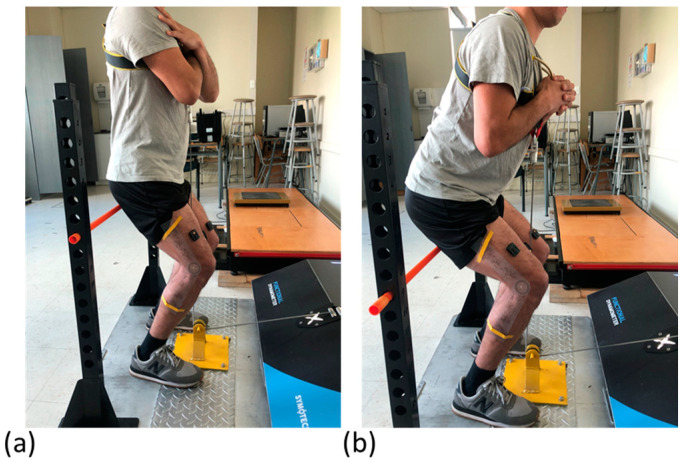
Initial positions for measuring the resisted plyometric exercise without jumping in a representative male participant. In (**a**), the knee joints are bent at 60°, while in (**b**), the knee joints are bent at 90°.

Lower limb strength, power, and work were evaluated using a functional electromechanical dynamometer (FEMD) (DynaSystem, Model Research, Spain) with a precision of 3 mm for displacement, 100 g for detected loads, a sampling frequency of 1000 Hz, and a speed range between 0.05 and 2.80 m/s. The resisted plyometric exercise without jumping involved an initial position with the participants seated with knee joints bent at two different starting positions: 60° (Figure 1a) and 90° (Figure 1b). The assessment encompassed an isokinetic mode at speeds of 5 cm/s (to determine maximum voluntary contraction) and a tonic mode at 80% of body weight (for resisted plyometric exercise without jumping). Both modalities have demonstrated high reliability [10].

During the test, participants marked a consistent foot position on the floor to ensure repetition consistency. A secure vest was worn, where the dynamometer was attached at the xiphoid process. Participants completed three trials in the isokinetic mode and 20 trials in the isotonic mode (i.e., 10 at a knee starting position of 60° and 10 at 90°), with instructions to maintain hands crossed against the chest and perform rapid squats without lifting their feet off the floor. Participants were verbally encouraged to exert maximum effort and speed using standardized guidelines. A Matlab R2013a (MathWorks Inc., Natick, MA, USA) script was used to automatically select the 3 best concentric–eccentric repetitions (higher values) and calculate their mean, normalized to body weight. Consequently, lower limb outcomes included mean strength (kg), mean power (W/kg), and mean work (J/kg).

#### 2.3.3. Surface Electromyography (sEMG) (Figure 2)

Considering their significance for knee stability, sEMG activity of the vastus medialis (VM) and vastus lateralis (VL) was assessed using the Trigno Wireless System (Delsys, Natick, MA, USA). Prior to testing, skin preparation included shaving, abrasion, and alcohol cleaning, followed by the application of the Trigno Flex sensor, featuring a sample rate of 1950 Hz for sEMG signals and 148 Hz for the accelerometer. Electrode placement followed the “Surface Electromyography for the Noninvasive Assessment of Muscles” (SENIAM) recommendations http://www.seniam.org (accessed on 20 March 2023) for the D and ND leg and was secured with 3M adhesive tape (3M, Canada). The sEMG signals were then amplified (input impedance 120 kΩ, signal-to-noise ratio 750, interelectrode distance 10 mm) within a gain range of 500–5000 and transmitted wirelessly to a computer through the Trigno Base Station. The sEMG data were processed using EMGworks^®^ software (Delsys, Natick, MA, USA) and filtered with a 10 Hz high-pass and 500 Hz low-pass second-order infinite impulse response Butterworth filter. For sEMG data analysis during the exercises, the root mean square (RMS) method was employed, applying a 60 ms moving window to calculate RMS values, in accordance with established practices [11].

During the resisted plyometric exercise without jumping, sEMG of the muscles was concurrently recorded with the acceleration detected by the Trigno Flex sensor located on the femur’s greater trochanter. A pilot session was conducted to assess the acceleration signals during the exercise, leading to the identification of an initial peak corresponding to the initiation of the exercise repetition and another peak observed at the conclusion of the exercise repetition. The RMS sEMG data were expressed as a percentage of the maximum voluntary contraction (MVC) [12] utilizing the highest sEMG recorded during the resisted plyometric without jumping trials at 5 cm/s [13].

**Figure 2:**
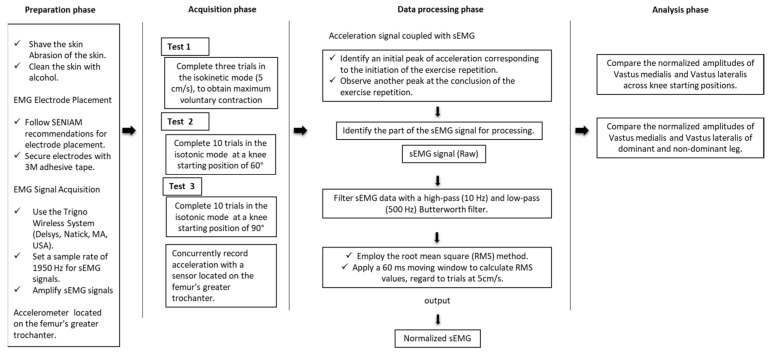
Flow chart diagram of sEMG recording procedures.

### 2.4. Statistical Analyses

The normality of the data was confirmed using the Shapiro–Wilk test (*p* > 0.05). As the majority of the data were non-normally distributed, they are presented as medians and interquartile ranges. However, since mean power was normally distributed, these data are presented as means ± standard deviations. The analyses of distinct aspects, including initial knee positions, D and ND legs, and comparisons between concentric and eccentric phases, were conducted independently. Parametric data were assessed using a paired *t*-test, while nonparametric data were analyzed using the Wilcoxon matched-pairs test. These analyses did not treat these factors as interrelated variables; rather, they were examined separately. Spearman rank correlation coefficient (*rs*) for the Wilcoxon matched-pairs test and partial eta squared (^η^_p_^2^) for the paired t-test were used to evaluate the effect size. Statistical significance was set at *p* ≤ 0.05, and all statistical analyses were performed using GraphPad Prism 8 (version 8.0.1).

## 3. Results

**Figure 3:**
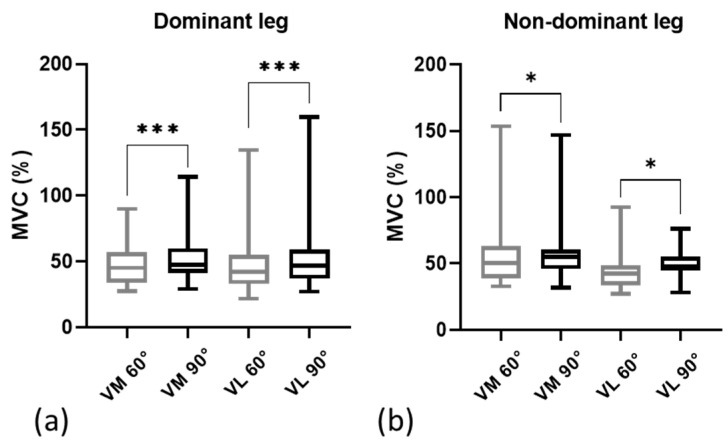
Vastus muscle RMS sEMG amplitudes between the resisted plyometric exercise without jumping at 60° and 90° of knee flexion: (**a**) comparison in the dominant leg; (**b**) comparison in the nondominant leg. Data are expressed as the median, and the bottom and top edges of the box indicate the 25th and 75th percentiles, respectively. MVC = maximum voluntary contraction; *** = *p* ≤ 0.001; * = *p* ≤ 0.05.

**Figure 4:**
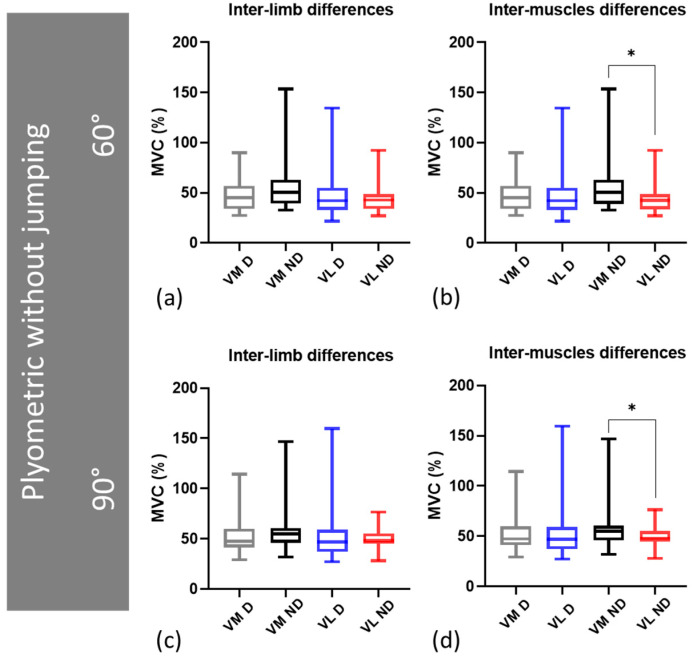
Interlimb and intermuscle comparison of vastus muscle RMS sEMG activity: (**a**,**c**) Interlimb differences at 60° and 90°, respectively; (**b**,**d**) intermuscle differences at 60° and 90°, respectively. Data are expressed as the median, and the bottom and top edges of the box indicate the 25th and 75th percentiles, respectively. MVC = maximum voluntary contraction; * = *p* ≤ 0.05.

The comparison of RMS sEMG between the plyometric exercise without jumping at 60° and 90° of knee flexion is presented in Figure 3. The results showed significantly higher values in D VM 47.4%, VL 46.9% at 90° (Figure 3a, Table 1), and ND VM 54.8%, VL 48.1% (Figure 3b, Table 1). Additionally, the interlimb comparison of vastus muscle RMS sEMG (i.e., between D and ND leg) revealed no significant differences (Figure 4a,c, Table 2). However, the intermuscle differences (i.e., between VM and VL) revealed higher values at both 60° (VM 50.4%) and 90° (VM 54.8%) exclusively in the ND leg (Figure 4b,d, Table 2).

**Figure 5:**
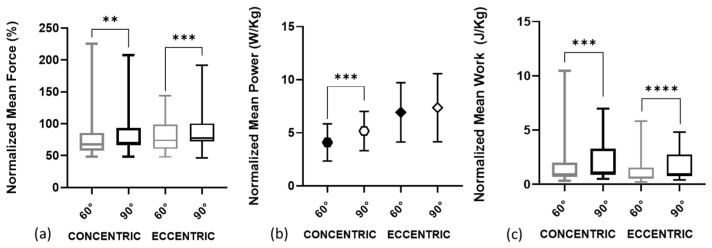
Comparisons of strength, power, and work between the two knee positions during the concentric and eccentric phases of the resisted plyometric exercise without jumping: (**a**) mean force; (**b**) mean power; (**c**) mean work. Mean force and mean work are expressed as the median, and the bottom and top edges of the box indicate the 25th and 75th percentiles, respectively. Mean power is expressed as mean ± standard deviation. *** = *p* ≤ 0.001; ** = *p* ≤ 0.05; **** = *p* ≤ 0.0001.

The comparison of strength, power, and work between the two knee positions (i.e., 60° and 90°) during the concentric and eccentric phases of the resisted plyometric exercise without jumping is presented in Figure 5. Significantly higher differences at 90° were observed in mean force 69.9% (Figure 5a, Table 3), mean power 5.2 ± 1.8 W/kg (Figure 5b, Table 3), and mean work 1.1 J/kg (Figure 5c, Table 3) during the concentric phase, and in mean force 77.5% (Figure 5a, Table 3), and mean work 0.9 J/kg (Figure 5c, Table 3) during the eccentric phase.

## 4. Discussion

This study aimed to (i) investigate the effects of resisted plyometric exercise without jumping at two knee flexion positions (60 and 90 degrees) on the muscle activity of the vastus muscles with regard to limb dominance and (ii) assess strength, power, and work during the concentric–eccentric phases of resisted plyometric exercise without jumping. The results revealed a significantly higher level of muscle activity in the VM and VL muscles when the exercises were performed at a 90-degree knee flexion position in both the D and ND legs. Additionally, significant intermuscle differences were observed at both 60° and 90° knee flexion positions, with higher values recorded for VM in the ND leg. Regarding strength, power, and work metrics, significantly higher values were observed when initiating the exercises from a 90-degree knee flexion position. This encompassed concentric mean strength, mean power, and mean work, as well as eccentric mean strength and mean work. These findings offer valuable insights into the influence of knee flexion position on muscle activity and performance during resisted plyometric exercises without jumping. This strategy aims to harness the SSC by training muscles to rapidly stretch and contract forcefully, resulting in significant improvements in explosive strength and reactive ability, all without the need for jumps. This approach reduces joint impact, which may have practical implications for designing tailored training programs and optimizing lower limb muscle engagement and output.

Study results demonstrated notable variations in muscle activity between 60°and 90° positions, particularly in favor of the 90° knee flexion position. These results are in line with prior research [14,15], which observed similar trends in muscle activation during squat exercise at different depths. The observed intermuscle differences between VM and VL were also consistent with previous literature, underscoring the intricate interplay between these muscle groups [15,16,17]. The higher muscle activation observed at 90° of knee flexion can be attributed to the increased demand for force production. At 90° of knee flexion, the lever arm for the subject’s body weight is greater than at 60° of knee flexion, resulting in a higher knee flexion torque. Consequently, more motor units are recruited in the vastus medialis and lateralis to generate the necessary force for this task, leading to the higher muscle activity levels observed. These findings underscore the significance of incorporating a 90-degree knee flexion angle when designing resisted plyometric exercises without jumping. Therefore, for rehabilitation exercises, it can be advantageous to include exercises that commence at a 90° knee flexion angle. This approach can aid individuals with vastus muscle inhibition in building knee stability and strength, which is essential for successful rehabilitation, as optimal muscle activation plays a pivotal role in overall performance. However, in addressing the scope of this investigation, it is important to note that deriving necessary adjustments to training and rehabilitation programs goes beyond the focus of this study. The determination and implementation of such adjustments fall within the domain of medical professionals.

The observed differences in muscle activity between the VM and VL at both 60° and 90°, particularly in the ND leg, may be attributed to muscle architecture and/or neuromuscular factors. The VM and VL possess distinct muscle architectures, with differences in fiber orientation and attachment points. The VM, for instance, has a more oblique fiber arrangement compared to the VL, which may result in varying mechanical advantages and force production capabilities at different knee flexion angles [18,19,20]. These architectural disparities can lead to variations in muscle activation patterns during dynamic movements. On the other hand, neuromuscular control plays a pivotal role in muscle activation patterns. Differences in motor unit recruitment strategies between the VM and VL can account for the observed intermuscle differences. The central nervous system modulates motor unit firing rates based on task demands [21] and muscle function [22]. Thus, the VM and VL may exhibit distinct motor unit recruitment patterns during the resisted plyometric exercise without jumping, contributing to differences in muscle activity. Furthermore, asymmetries in muscle activation between the D and ND side are commonly observed in the literature [9,23,24]. These differences may stem from variations in limb dominance and coordination patterns. The nondominant (ND) leg may exhibit greater differences in vastus medialis (VM) and vastus lateralis (VL) activation due to varying neuromuscular adaptations resulting from everyday activities and functional demands. Additionally, VM and VL are biomechanically interconnected muscles that work together to control knee joint stability and movement [25,26]. Given that the nondominant leg is primarily responsible for stabilization during bipedal movements [14], this implies that during resisted nonjump plyometric exercises, the nondominant limb may require increased stabilization, resulting in higher vastus medialis activity, regardless of the knee flexion angle. In summary, the intermuscle differences observed between VM and VL during a resisted plyometric exercise without jumping at different knee flexion angles can be attributed to a complex interplay of muscle architecture and neuromuscular control. Coaches and trainers should consider incorporating exercises with a 90° knee flexion angle to maximize muscle activation, particularly in the VM and VL. This may be particularly relevant to improve lower limb strength and power. Furthermore, understanding the intermuscle differences emphasizes the necessity of a balanced training program that targets both the VM and VL, as imbalances between these muscles can contribute to knee instability [27]. Furthermore, if there is a need to increase the muscle activity of the vastus medialis in the nondominant leg to enhance stability during bipedal functional movements in rehabilitation programs, it is recommended to incorporate resisted nonjump plyometrics at 60° or 90° knee flexion angles. This approach can contribute to a more efficient and effective recovery process.

Regarding the impact of knee flexion angle on strength, power, and work during both the concentric and eccentric phases of a resisted plyometric exercise without jumping, the results demonstrated a significant advantage for the 90° knee flexion position across multiple parameters, including mean force, mean power, and mean work during both phases. These findings align with the trends observed in previous studies [28,29], which have reported increased force production and power generation at greater knee flexion angles. Additionally, our results are consistent with the biomechanical principles governing muscle contraction, suggesting that the length–tension relationship and muscle architecture play pivotal roles in force and power output [30]. Indeed, a greater knee flexion angle enhances the length–tension relationship of the muscle fibers, enabling them to generate force more effectively [31,32]. This mechanical advantage achieved at 90° knee flexion led to improved power generation and work output. Furthermore, at 90° of knee flexion, the patella’s position enhances the leverage and mechanical advantage of the quadriceps muscles, including the vastus medialis and lateralis. This improved leverage allows the muscles to generate more force.

The concentric and eccentric phases of muscle contraction each exhibit distinct biomechanical characteristics. During the concentric phase, the observed increases in mean force, mean power, and mean work at 90° knee flexion suggest that this angle offers a biomechanically favorable starting position for force generation. Conversely, during the eccentric phase, the increased mean force and mean work at 90° may indicate enhanced force absorption and control, which are crucial for eccentric muscle actions [33]. Our findings have significant relevance for exercise prescription, training regimens, and rehabilitation programs. Coaches and trainers can optimize training protocols by incorporating resisted plyometric exercises without jumping with a 90° knee flexion angle to maximize force, power, and work output. Furthermore, rehabilitation programs for individuals recovering from lower limb injuries may strategically utilize the 90° knee flexion position to enhance force absorption and control, which are vital components of injury prevention and rehabilitation [34]. This knowledge provides a valuable framework for tailoring resisted plyometric exercise without jumping interventions to specific performance and rehabilitation goals, thereby facilitating more effective and efficient training and recovery.

Certain limitations must be addressed. In the initial instance, it is imperative to acknowledge the bilateral evaluation of strength, power, and work, which imposes constraints on the potential for comparative analysis across limbs. To mitigate this constraint, forthcoming investigations may contemplate incorporating metrics that discriminate between unilateral and bilateral assessments of strength, power, and work. Secondly, the absence of a sex-based analysis among study subjects has the potential to restrict the extent to which the study’s conclusions can be extrapolated. An alternative course of action would involve conducting a sex-stratified analysis to systematically explore any divergences in outcomes, given that the available data are insufficient for deriving such conclusions. It is important to note that the normalization to body weight aimed to abstract from sex-specific effects.

## 5. Conclusions

Our findings suggest that initiating resisted plyometric exercises without jumping at a 90° knee flexion position results in significantly increased muscle activity in both the VM and VL muscles, regardless of limb dominance. Moreover, we observed substantial intermuscle differences, with the VM in the ND leg displaying higher activation levels at both 60° and 90° knee flexion positions. Furthermore, our investigation revealed that starting the exercises from a 90° knee flexion position led to significantly greater levels of strength, power, and work during the concentric–eccentric phases of the resisted plyometric exercise without jumping. These findings emphasize the importance of knee flexion position in tailoring resisted plyometric without jumping training programs to optimize muscle engagement and functional performance.

## Figures and Tables

**Table 1:** Muscle RMS sEMG amplitude differences (MVC %) during plyometric exercise without jumping at 60° and 90° knee flexion.

Variable	Leg	Muscle	60°	90°	*p*	ES
MVC %	Dominant	VM	45.1 (33.8–56.9)	47.4 (41.2–59.8)	0.0008 ***	0.90
		VL	42.0 (32.9–55.0)	46.9 (37.1–59.0)	0.0004 ***	0.86
MVC %	Nondominant	VM	50.4 (39.0–63.0)	54.8 (45.8–60.6)	0.047 *	0.88
		VL	42.5 (33.7–48.5)	48.1 (44.9–55.2)	0.021 *	0.67

Values are expressed as median and interquartile range. Significant differences, * = *p* ≤ 0.05; *** = *p* < 0.0001. Vastus medialis (VM), vastus lateralis (VL), maximum voluntary contraction (MVC), and effect size (ES).

**Table 2:** Intermuscles amplitude differences at 60° and 90° knee flexion.

Variable	Leg	Knee Angle	VM	VL	*p*	ES
MVC %	Dominant	60°	45.1 (33.8–56.9)	42.0 (32.9–55.0)	0.811	0.32
		90°	47.4 (41.2–59.8)	46.9 (37.1–59.0)	0.696	0.40
MVC %	Nondominant	60°	50.4 (39.0–63.0)	42.5 (33.7–48.5)	0.003 *	0.56
		90°	54.8 (45.8–60.6)	48.1 (44.9–55.2)	0.005 *	0.62

Values are expressed as median and interquartile range. Significant differences, * = *p* ≤ 0.05. Vastus medialis (VM), vastus lateralis (VL), maximum voluntary contraction (MVC), and effect size (ES).

**Table 3:** Comparison of strength, power, and work between the two knee positions during the concentric and eccentric phases of the resisted plyometric exercise without jumping.

Variable	Phase	60°	90°	*p*	ES
Mean force (%)	Concentric	67.4 (57.5–85.6)	69.9 (66.3–93.2) **	0.004	0.82
	Eccentric	73.8 (60.7–98.8)	77.5 (72.3–100.2) ***	0.0006	0.89
Mean power (W/kg)	Concentric	4.1 ± 1.7	5.2 ± 1.8 ***	0.0002	0.69
	Eccentric	6.9 ± 2.8	7.4 ± 3.2	0.44	0.84
Mean work (J/kg)	Concentric	0.9 (0.6–2.0)	1.1 (0.9–3.2) ****	<0.0001	0.92
	Eccentric	0.65 (0.5–1.5)	0.9 (0.7–2.8) ****	<0.0001	0.92

Mean force and mean work values are expressed as median and interquartile ranges. Mean power is expressed as mean ± standard deviation. Significant differences, ** = *p* ≤ 0.05; *** = *p* ≤ 0.001; **** = *p* ≤ 0.0001. Vastus medialis (VM), vastus lateralis (VL), and effect size (ES).

## Data Availability

The data presented in this study are available on request from the corresponding author. The data are not publicly available due to ethical committee restrictions.
